# Supplementary figures and images for: Introducing THOR, a Model Microbiome for Genetic Dissection of Community Behavior
Source: mBio. 2019 Mar 5;10(2):e02846-18. doi: 10.1128/mBio.02846-18 (PMC6401489; doi:10.1128/mBio.02846-18)

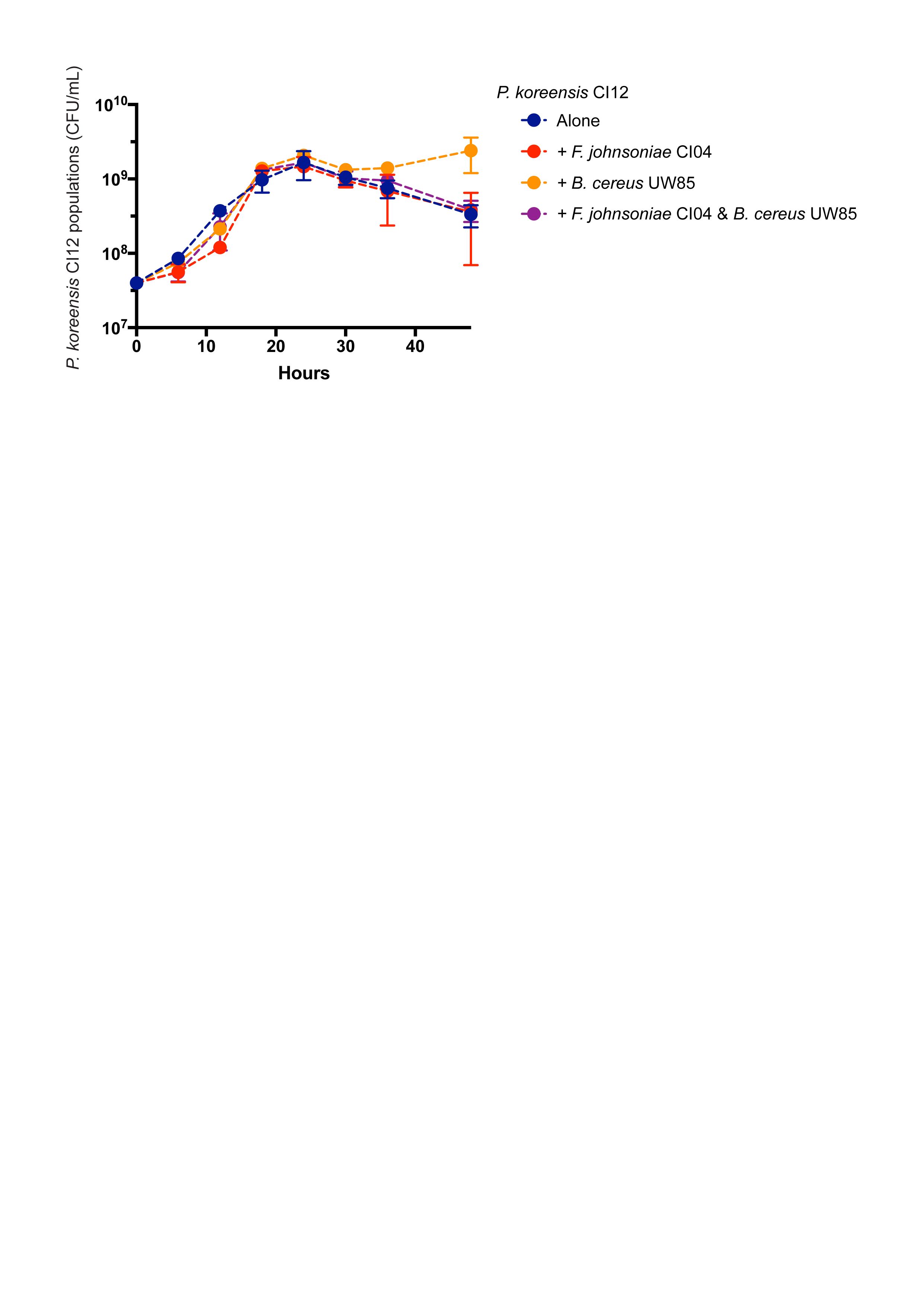

Supplement: FIG S1 [file mBio.02846-18-sf001.tif]

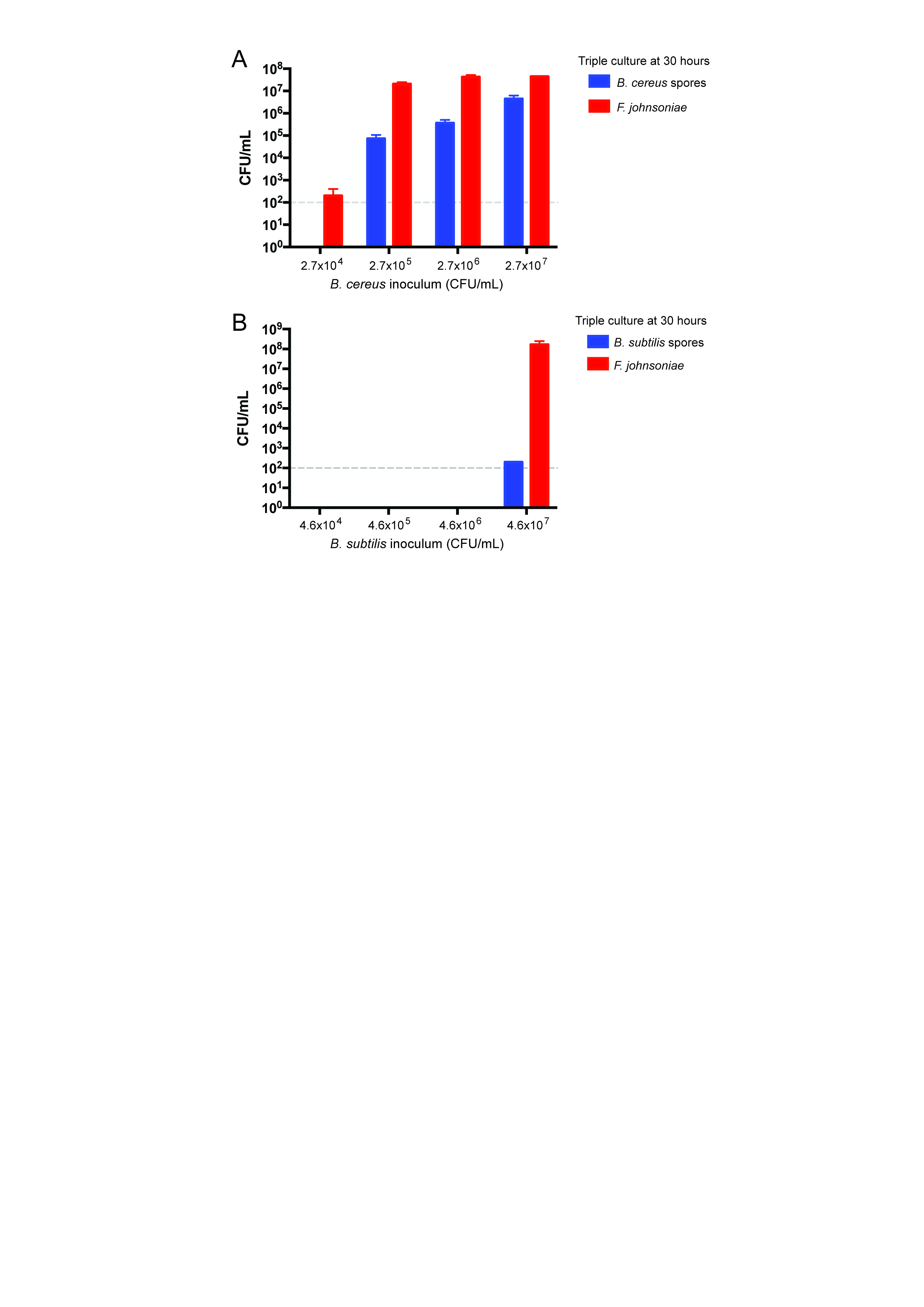

Supplement: FIG S2 [file mBio.02846-18-sf002.tif]

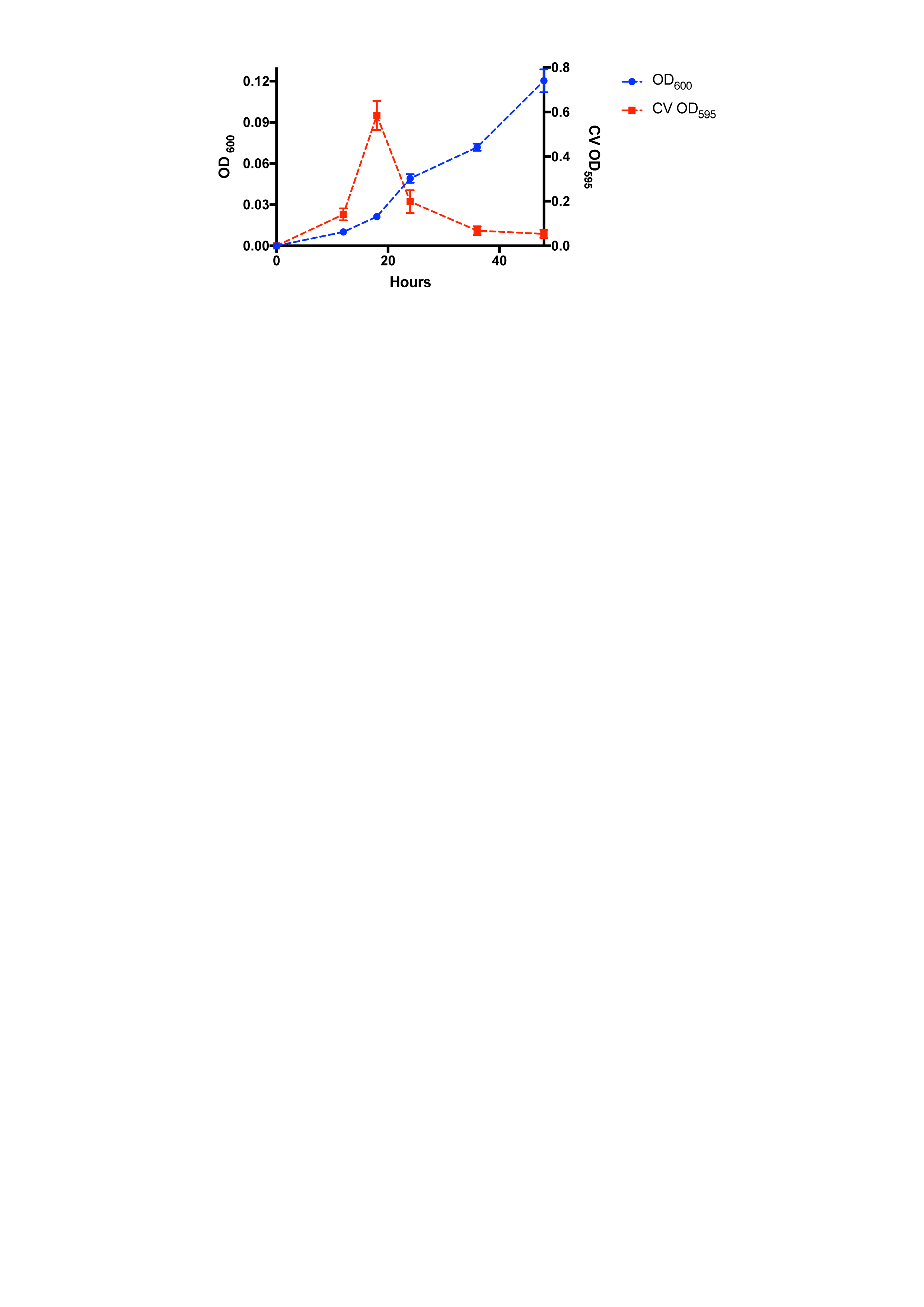

Supplement: FIG S3 [file mBio.02846-18-sf003.tif]
