# Supplementary material for: Introducing THOR, a Model Microbiome for Genetic Dissection of Community Behavior
Source: mBio. 2019 Mar 5;10(2):e02846-18. doi: 10.1128/mBio.02846-18 (PMC6401489; doi:10.1128/mBio.02846-18)
Supplement: TABLE S1 [file mBio.02846-18-st001.docx]

**TABLE S1.** Area of *B. cereus* colonies is larger in the presence of either *F. johnsoniae* or *P. koreensis.*

|  | Area *B. cereus* colony (cm^2^) | |
| --- | --- | --- |
|  | Day 2 | Day 5 |
| *B. cereus* alone | 1.3 ± 0.06 | 6.3 ± 0.42 |
| *B. cereus* with *F. johnsoniae* | 3.0 ± 0.24 | 24.3 ± 0.55 |
| *B. cereus* with *P. koreensis* | 1.8 ± 0.2 | 23.8 ± 2.66 |
