# Supplementary material for: Introducing THOR, a Model Microbiome for Genetic Dissection of Community Behavior
Source: mBio. 2019 Mar 5;10(2):e02846-18. doi: 10.1128/mBio.02846-18 (PMC6401489; doi:10.1128/mBio.02846-18)
Supplement: TABLE S2 [file mBio.02846-18-st002.docx]

**TABLE S2.** Strains used in this study

| Name | Phyla | Family | Abbreviation |
| --- | --- | --- | --- |
| *Sphingobacterium* sp. CI01 | Bacteroidetes | Sphingobacteriales | CI01 |
| *Chryseobacterium* sp. CI02 | Bacteroidetes | Flavobacteriales | CI02 |
| *Flavobacterium johnsoniae* CI04 | Bacteroidetes | Flavobacteriales | CI04 |
| *Pseudomonas* sp. CI14 | Proteobacteria | Pseudomonadales | CI14 |
| *Pseudomonas* sp. RI46 | Proteobacteria | Pseudomonadales | RI46 |
| *Pseudomonas koreensis* CI12 | Proteobacteria | Pseudomonadales | CI12 |
| *Stenotrophomonas maltophilia* CI13 | Proteobacteria | Xanthomonadales | CI13 |
| *Lysobacter* sp. RI17 | Proteobacteria | Xanthomonadales | RI17 |
| *Serratia* sp. CI20 | Proteobacteria | Enterobacteriales | CI20 |
| *Pantoea agglomerans* sp. RI11 | Proteobacteria | Enterobacteriales | RI11 |
| *Achromobacter* sp. CI16 | Proteobacteria | Burkholderiales | CI16 |
| *Variovorax* sp. CI17 | Proteobacteria | Burkholderiales | CI17 |
| *Stenotrophomonas maltophilia* RI33 | Proteobacteria | Burkholderiales | RI33 |
| *Delftia acidovorans* CI11 | Proteobacteria | Burkholderiales | CI11 |
| *Agrobacterium tumefaciens* RI12 | Proteobacteria | Rhizobiales | RI12 |
| *Ochrobactrum* sp. RI54 | Proteobacteria | Rhizobiales | RI54 |
| *Arthrobacter* sp. CI34 | Actinobacteria | Actinomycetales | CI34 |
| *Microbacterium* sp. CI59 | Actinobacteria | Actinomycetales | CI59 |
| *Microbacterium* sp. RI03 | Actinobacteria | Actinomycetales | RI03 |
| *Paenibacillus* sp. RI40 | Firmicutes | Bacillales | RI40 |
| *Bacillus cereus* UW85 | Firmicutes | Bacillales | UW85 |
| *Sphingobacterium* sp. CI48 | Bacteroidetes | Sphingobacteriales | CI48 |
| *Chryseobacterium* sp. CI26 | Bacteroidetes | Flavobacteriales | CI26 |
| *Flavobacterium johnsoniae* CI64 | Bacteroidetes | Flavobacteriales | CI64 |
| *Bacillus subtilis* NCIB3160 | Firmicutes | Bacillales | NCIB3160 |
