# Supplementary material for: Introducing THOR, a Model Microbiome for Genetic Dissection of Community Behavior
Source: mBio. 2019 Mar 5;10(2):e02846-18. doi: 10.1128/mBio.02846-18 (PMC6401489; doi:10.1128/mBio.02846-18)
Supplement: TABLE S3 [file mBio.02846-18-st003.docx]

**TABLE S3.** Primers used in this study.

| Name | Sequence |
| --- | --- |
| mut_spo0HA1 | CACCGGATCCGCCAATTGGGTTAGTAATTGGTAGTGAAGG |
| mut_spo0HA2 | GACAATGTCAATGACATGCATTTTGTACTGTCCTTGATCCCTCCGACCGCTATTTATTTAG |
| mut_spo0HB1 | CTAAATAAATAGCGGTCGGAGGGATCAAGGACAGTACAAAATGCATGTCATTGACATTGTC |
| mut_spo0HB2 | GGATCCACGTACAACATACCAAGAATCATCAGTCATG |
